# A Peculiar Case

**Published:** 1871-03

**Authors:** 


### A PECULIAR CASE.

Dr. H. Scott, of Lancaster, O., writes that he found a remarkable freak of nature practiced on the mouth of a young man of German extraction, who called at his office to have some teeth filled. On the right side above there were two perfect central incisors almost alike, thus making five perfect incisors between the cuspids. The cuspids, bicuspids, and two molars on the same side, were all normal, but correspondingly thrown back, and the third molar was wanting. The centrals both above and below were also normal, and there were no other departures from regularity. The inferior wisdom teeth were fully developed, as also the left superior. The odd tooth is simply supernumerary, but it was quite equal to either of the other centrals in size and symmetry.

